# Postoperative Complications of Laparoscopic Total Gastrectomy versus Open Total Gastrectomy for Gastric Cancer in a Meta-Analysis of High-Quality Case-Controlled Studies

**DOI:** 10.1155/2016/2617903

**Published:** 2016-11-30

**Authors:** Mikito Inokuchi, Sho Otsuki, Norihito Ogawa, Toshiro Tanioka, Keisuke Okuno, Kentaro Gokita, Tatsuyuki Kawano, Kazuyuki Kojima

**Affiliations:** ^1^Department of Gastrointestinal Surgery, Tokyo Medical and Dental University, 1-5-45 Yushima, Bunkyo, Tokyo 113-8519, Japan; ^2^Department of Minimally Invasive Surgery, Tokyo Medical and Dental University, 1-5-45 Yushima, Bunkyo, Tokyo 113-8519, Japan

## Abstract

*Background*. Some meta-analyses of case-controlled studies (CCSs) have shown that laparoscopic or laparoscopy-assisted total gastrectomy (LTG) had some short-term advantages over open total gastrectomy (OTG). However, postoperative complications differed somewhat among the meta-analyses, and some CCSs included in the meta-analyses had mismatched factors between LTG and OTG.* Methods*. CCSs comparing postoperative complications between LTG and OTG were identified in PubMed and Embase. Studies matched for patients' status, tumor stage, and the extents of lymph-node dissection were included. Outcomes of interest, such as anastomotic, other intra-abdominal, wound, and pulmonary complications, were evaluated in a meta-analysis performed using Review Manager version 5.3 software.* Result*. This meta-analysis included a total of 2,560 patients (LTG, 1,073 patients; OTG, 1,487 patients) from 15 CCSs. Wound complications were significantly less frequent in LTG than in OTG (*n* = 2,430; odds ratio [OR] 0.30, 95% confidence interval [CI] 0.29–0.85, *P* = 0.01, *I* ^2^ = 0%, and OR 0.46, 95% CI 0.17–0.52, *P* < 0.0001, *I* ^2^ = 0%). However, the incidence of anastomotic complications was slightly but not significantly higher in LTG than in OTG (*n* = 2,560; OR 1.44, 95% CI 0.96–2.16, *P* = 0.08, *I* ^2^ = 0%).* Conclusion*. LTG was associated with a lower incidence of wound-related postoperative complications than was OTG in this meta-analysis of CCSs; however, some concern remains about anastomotic problems associated with LTG.

## 1. Introduction

Laparoscopic (laparoscopy-assisted) distal gastrectomy (LDG) is an established minimally invasive procedure for the treatment of gastric cancer, especially in Eastern Asia. However, whether laparoscopic (laparoscopy-assisted) total gastrectomy (LTG) can be used as a standard treatment remains controversial. One of the reasons is that advanced techniques are required to perform lymph-node dissection along the splenic artery as well as reconstruction by esophagojejunostomy. Another reason is that the incidence of gastric cancer is lower in the upper portion of the stomach than in the middle or lower portions, especially in Eastern Asia. To our knowledge, a randomized controlled trial (RCT) comparing LTG with conventional open total gastrectomy (OTG) has yet to be performed. Six meta-analyses of case-controlled studies (CCSs) comparing LTG with OTG showed several short-term advantages of LTG, such as a lower operative bleeding volume, earlier bowel movement, earlier oral intake, less pain, and a shorter hospital stay [1–6]. However, the reliability of these potential advantages of LTG was poor owing to the high statistical heterogeneity in these meta-analyses, which cannot be ignored. Postoperative complications were less common after LTG, and the heterogeneity of these complications was very low in previously reported meta-analyses. In some CCSs included in published meta-analyses, patient groups were mismatched for age, gender, tumor stage, or anastomotic procedure, which might have had an effect on the comparison of postoperative complications between LTG and OTG. One meta-analysis revealed a significantly lower incidence of surgical complications in LTG than in OTG [1], while another meta-analysis showed a significantly lower incidence of medical complications in LTG [2]. Moreover, a multi-institutional CCS matched on the basis of propensity score revealed the disadvantage of anastomotic complications in LTG [7].

To clarify differences in categorized postoperative complications between LTG and OTG, we conducted an updated meta-analysis that included CCSs in which age, gender, physical status, tumor stage, extent of lymph-node dissection, and reconstruction procedure were matched.

## 2. Materials and Methods

### 2.1. Study Selection

A search of studies published from January 1994 through July 2016 was carried out in the PubMed and Embase databases. The search terms included “laparoscopic,” “total gastrectomy,” and “gastric cancer.” The meta-analysis included CCSs that compared postoperative complications between LTG and OTG. The following types of studies were excluded before review of the full texts: (a) reports in languages other than English, (b) studies not comparing LTG with OTG, and (c) reports of studies that were not available online. In addition, the following studies were excluded after review of the full texts: (d) reviews or meta-analyses and (e) studies not showing complications. To minimize bias in this meta-analysis of postoperative complications, we excluded studies that met any of the following conditions: (f) including robotic surgery, (g) including D0 lymphadenectomy, (h) including less than 10 patients in one group, (i) including patients who received neoadjuvant chemotherapy, and (j) showing a significant statistical difference between LTG and OTG in at least one of the following variables: age, gender, body mass index (BMI), the rate of comorbidity, American Society of Anesthesiologists physical status (ASA-PS), pathological tumor stage, extent of lymph-node dissection, or reconstruction method. However, some studies showing no statistical difference between LTG and OTG in several of these factors were included in the present meta-analysis.

### 2.2. Quality Assessment

The Newcastle-Ottawa scoring system (NOS) was used to assess the quality of CCSs [8]. In the NOS, the maximum evaluation was four stars for selection, two for comparability, and three for outcome assessment.

### 2.3. Outcomes of Interest

On the basis of the results of a previous multi-institutional CCS [7], LTG and OTG were compared with regard to the following postoperative complications: (a) anastomotic complications, including anastomotic leakage or stump leakage, anastomotic stenosis, and anastomotic bleeding; (b) other intra-abdominal complications, including conditions such as intra-abdominal abscess, intra-abdominal bleeding, pancreatic fistula, pancreatitis, ileus, internal hernia, and cholecystitis; (c) wound complications, including wound infection and wound seroma; and (d) pulmonary complications, including pneumonia, pulmonary infection, and pleural effusion. Mortality was also compared between the groups.

### 2.4. Statistical Analysis

Review Manager version 5.3 (Cochrane Collaboration, Oxford, UK) was used to perform this meta-analysis. For categorical variables, postoperative complications were extracted from the trial report; odds ratio (OR) was calculated on the basis of the total number of patients and the observed number of events of interest in all groups, using a random-effect model. In the tables summarizing our results, squares indicate point estimates of OR, with 95% confidential intervals (CI) indicated by horizontal bars. The diamond represents the summary OR with 95% CI from the included studies. *P* values of < 0.05 were considered to indicate statistical significance.

The *I* ^2^ statistic was used to quantitatively assess heterogeneity. In addition, graphical exploration with funnel plots was used to evaluate publication bias.

## 3. Results

### 3.1. Selected Studies

**Figure 1:**
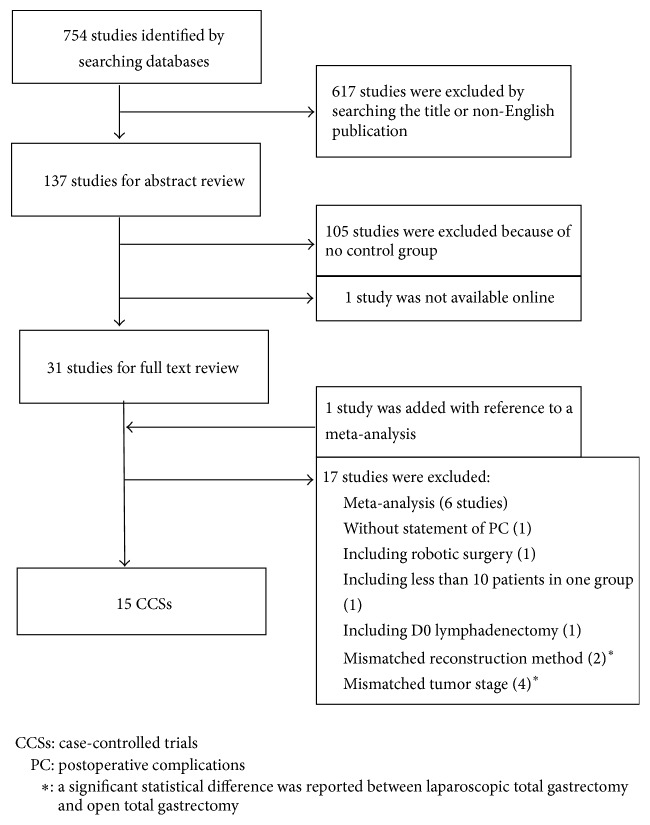
Flowchart of study selection.

A flowchart summarizing the study selection process is shown in Figure 1. Of 754 publications that were identified by the above search, 137 publications were selected by checking the title. One hundred five of these publications not including a control group were unrelated to our purpose and were therefore excluded after screening the abstract. The full text of 1 publication was not available online. The full texts were available for the remaining 31 articles, and 1 publication was added because it was included as a reference in the report of another meta-analysis. Finally, 15 CCSs [7, 9–22] met the inclusion criteria for this meta-analysis, and all were published between 2008 and 2015. The quality assessment of these CCSs is shown in Table 1. In the results of assessment, the study by Guan et al. had one star for comparability because whether age differed significantly between LTG and OTG was not reported.

The details of the included studies are shown in Table 2. This meta-analysis included a total of 2,560 patients, among whom 1,073 underwent LTG and 1,487 underwent OTG. Male and female patients were, respectively, 668 and 332 in the LTG group and 946 and 484 in the OTG group; however, sex was unknown for 73 patients who underwent LTG and 57 who underwent OTG in 2 studies. All patients underwent D1+ to D2 lymphadenectomy, and 3 studies included patients who underwent splenectomy. As for the pathological findings of tumors, 481 and 822 patients had early cancer, and 251 and 330 patients had advanced cancer in the LTG group and OTG group, respectively. The pathological tumor stage was reported for 525 of the other 676 patients in whom tumor depth was not specified, and no pathological findings were reported for 151 patients in 2 studies.

### 3.2. Outcomes of Interest

**Figure 2:**
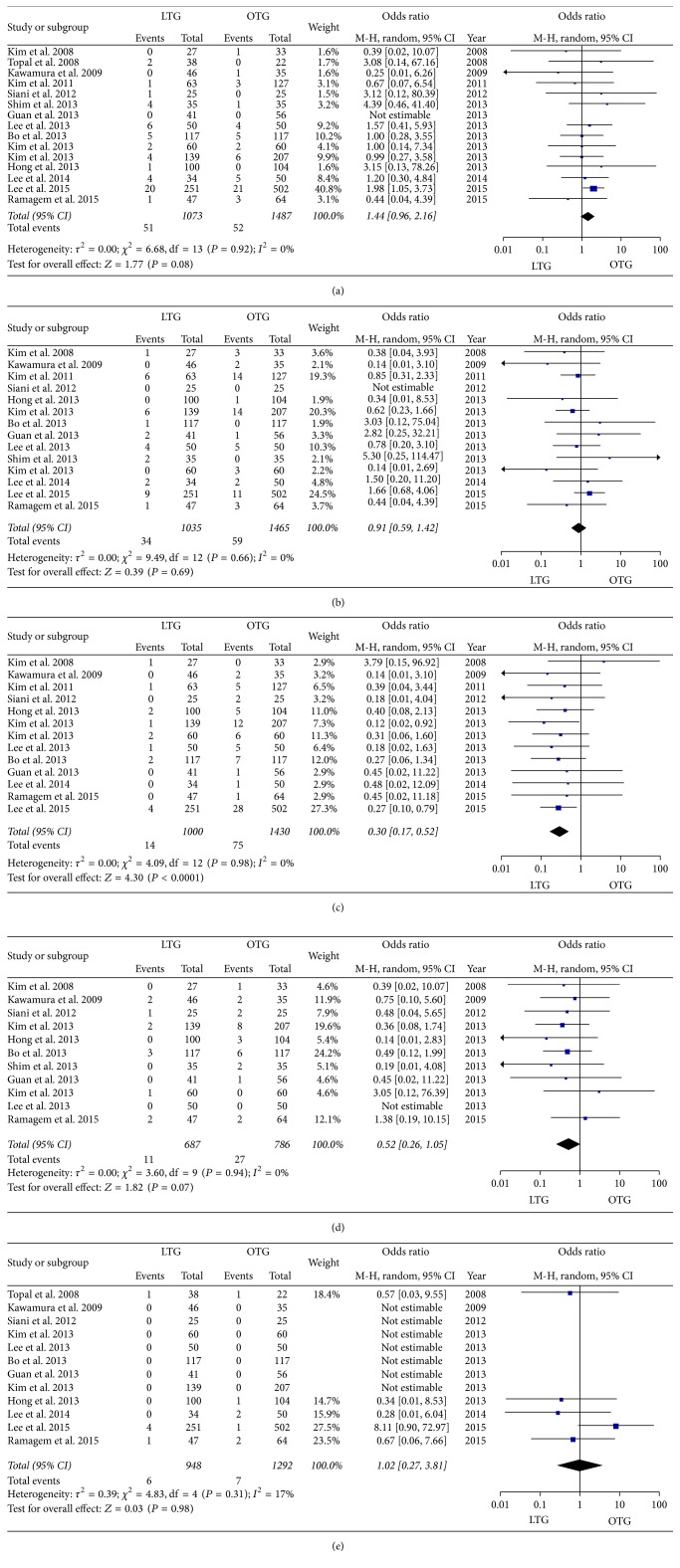
(a) Comparison of anastomotic complications between LTG and OTG. (b) Comparison of other intra-abdominal complications between LTG and OTG. (c) Comparison of wound complications between LTG and OTG. (d) Comparison of pulmonary complications between LTG and OTG. (e) Comparison of mortality between LTG and OTG.

Anastomotic complications were reported in 103 of the 2,560 patients from the 15 studies. The incidence of anastomotic complications was slightly but not significantly higher in the LTG group than in the OTG group (*n* = 2,560, odds ratio [OR] 1.44, 95% confidence interval [CI] 0.96–2.16, *P* = 0.08, *I* ^2^ = 0%) (Figure 2(a)). Thirty-four patients were given a diagnosis of anastomotic leakage (17 in LTG and 17 in OTG), and the other 12 (6 in LTG and 6 in OTG), 12 (7 in LTG and 5 in OTG), and 5 (2 in LTG and 3 in OTG) patients were given diagnoses of stump leakage, anastomotic stenosis, and anastomotic bleeding, respectively. Forty patients (19 in LTG and 21 in OTG) were categorized as having anastomotic complications. Other intra-abdominal complications were reported in 93 of the total of 2,500 patients from the 14 studies and were similar in LTG and OTG (OR 0.91, 95% CI 0.59–1.42, *P* = 0.69, *I* ^2^ = 0%, Figure 2(b)). Intra-abdominal abscess or infection was found most frequently (30 patients). Bleeding and ileus (including internal hernia) were found in 18 patients each. Twenty patients were categorized as having intra-abdominal complications other than anastomotic complications. Wound complications were reported in 89 of a total of 2,430 patients in 13 studies, and the incidence of wound complications was significantly lower in LTG than that in OTG (OR 0.30, 95% CI 0.17–0.52, *P* < 0.0001, *I* ^2^ = 0%) (Figure 2(c)). Forty-four patients were given a diagnosis of wound infection, although 41 patients were categorized as having wound complications. Pulmonary complications were reported in 38 of 1,472 patients from 11 studies. LTG was associated with a slightly but not significantly lower incidence of pulmonary complications as compared with OTG (OR 0.52, 95% CI 0.26–1.05, *P* = 0.07, *I* ^2^ = 0%) (Figure 2(d)). Twenty-three patients were given a diagnosis of pneumonia or pulmonary infection, although 11 patients were categorized as having pulmonary complications. Mortality was reported in 13 patients among a total of 2,240 patients from 12 studies and was similar in the two groups (OR 1.02, 95% CI 0.27–3.81, *P* = 0.98, *I* ^2^ = 17%) (Figure 2(e)).

Next, we divided all studies included in the present meta-analysis into two groups based on the median year of the period of study to evaluate the relation between postoperative complications and the study period. Eight studies in which median year of the study period was between 2004 and 2007 were categorized as the former group [7, 9–11, 13, 14, 18, 19], and 7 studies in which the median year was between 2008 and 2011 were categorized as the latter group [12, 15–17, 20–22]. The incidence of anastomotic complications in LTG was 5.9% (36/614) and 3.3% (15/459) in the former group and the latter group (*P* = 0.048), respectively. The incidence of anastomotic complications in OTG was 4.0% (34/844) and 3.6% (23/643) in the former group and the latter group, respectively (*P* = 0.65).

### 3.3. Publication Bias Assessment

Publication bias was assessed for each complication with the use of the funnel plots of the included studies. No complication was associated with publication bias, and a symmetric distribution was maintained (see Supplementary 1A-E in Supplementary Material available online at http://dx.doi.org/10.1155/2016/2617903).

## 4. Discussion

LTG was associated with a significantly lower incidence of wound complications than was OTG in this meta-analysis of CCSs, in which age, gender, physical status, tumor stage, extent of lymph-node dissection, and reconstruction procedure were matched between the two groups. In the present meta-analysis, many wound-related problems involved infection. In a meta-analysis of 15 CCSs reported by Xiong et al., the incidence of wound complications was significantly lower in LTG than in OTG [4]. Wang et al. reported two meta-analyses comparing LTG with OTG. One analysis included 18 CCSs and the other analysis included 9 CCSs of total gastrectomy with D2 lymphadenectomy. Only the incidence of wound infection was significantly lower in LTG than in OTG [5, 6]. According to the Centers for Disease Control and Prevention guidelines, there are patient-related and operation-related risk factors for surgical site infection [23]. The patient-related risks are age, obesity, nutritional status, diabetes, smoking, and length of the preoperative stay, and most of these factors were probably matched in the present meta-analysis. The operation-related risks are the duration of operation or the surgical technique, factors such as poor hemostasis, failure to obliterate dead space, and tissue trauma. Many of the studies included in the present meta-analysis reported less operative bleeding but a longer operative time in LTG than in OTG. In a meta-analysis of LDG that included matched CCSs, the incidence of wound infection was lower in LDG than in open distal gastrectomy (ODG) [24]. In a Korean RCT comparing LDG with ODG in patients with clinical stage I gastric cancer, LDG significantly reduced wound complications such as wound infection and dehiscence [25]. Available evidence thus suggests that a laparoscopic approach for gastric surgery might effectively decrease the risk of wound complications. However, a Chinese RCT comparing LDG with ODG in patients with advanced gastric cancer reported a similar incidence of wound problems [26]. The overall incidence of wound complications was only 1.1% (11/1,039) in both LDG and ODG in the Chinese RCT [26], whereas it was 5.3% (67/1,256, per protocol population) in the Korean RCT [25]. The concrete definitions of wound complications might have differed somewhat among studies. In the present study, the overall incidence of wound complications was 3.7% (89/2,430).

Anastomotic complications did not differ significantly between LTG and OTG in several previous meta-analyses [1–6]. However, a multi-institutional CCS in which the patients were matched by propensity score revealed a significantly higher rate of anastomotic complications in LTG [7]. In the present meta-analysis including CCSs, anastomotic complications were slightly but not significantly more common in LTG than in OTG. Anastomosis-related problems after LTG are an important concern, and many procedures for esophagojejunostomy after LTG have been developed [27, 28]. In a review article comparing different procedures for anastomosis after LTG, the use of circular staplers was significantly associated with higher incidences of both anastomotic leakage (4.7%) and stenosis (8.3%) as compared with the use of linear staplers (1.1% and 1.8%, resp.) [29]. One of the reasons might be that an anastomotic site formed by a linear stapler can probably secure a wider diameter than one formed by a circular stapler [29]. In a study of Orvil™ devices for esophagojejunostomy, the use of a smaller circular stapler (21 mm) was associated with a significantly higher incidence of anastomotic stenosis than was a normal-sized stapler (25 mm) [30]. The Japanese National Clinical Database (NCD) of digestive surgery reported that the incidence of anastomotic leakage after total gastrectomy was 4.4% (881/20,011) in 2011 [31]. In the present study, the overall incidence of anastomotic complications including leakage, stenosis, and bleeding was 4.0% (103/2,560) and did not differ from the incidence in the NCD. In the study by Lee et al., a high incidence of anastomotic complications after LTG (8.0%, 20/251) was obtained; however, patients treated since 2003 were included. Anastomotic complications and other postoperative outcomes are expected to improve in the future as surgeons acquire more experience and enhanced surgical skills. The incidence of postoperative complications after LTG was higher in the studies performed during the former period, whereas there was no difference in the incidence of postoperative complications after OTG between the former period and latter period in our study. In another study of the learning curve associated with LTG, experience performing LTG in approximately 45 or 100 patients was required to master the procedure [32, 33]. A prospective phase II study comparing LTG with laparoscopic proximal gastrectomy has begun in Japan, with anastomotic leakage as the primary endpoint, and that study is expected to provide conclusive evidence on anastomotic problems after LTG [34]. Obesity has been considered a contraindication for laparoscopic surgery because of technical difficulties and high conversion rates. In a previous study, the incidence of anastomotic complications was similar for laparoscopic gastrectomy and open gastrectomy in patients with a BMI of 30 kg/m^2^ or greater, although laparoscopic gastrectomy had a lower overall complication rate than open gastrectomy [35].

In the present meta-analysis, the incidence of postoperative pulmonary complications was slightly but not significantly lower in the LTG group than in the OTG group. Many pulmonary complications involved pneumonia in our study. These findings regarding pulmonary complications and pneumonia are supported by the results of other meta-analyses comparing LTG with OTG [3, 6]. In one meta-analysis comparing LDG with ODG, LDG reduced the risk of postoperative pneumonia slightly but not significantly [24]. Moreover, in a Korean RCT comparing LDG with ODG in patients with early gastric cancer, LDG was associated with a slightly but not significantly lower risk of pulmonary complications [25]. A Chinese RCT of LDG in patients with advanced cancer obtained a similar rate of pulmonary complications [26]. Total gastrectomy is one of the independent predictors of postoperative pulmonary complications, whereas distal gastrectomy is not [36]. Therefore, better-preserved respiratory function with less wound pain might more effectively prevent pulmonary complications in total gastrectomy than in distal gastrectomy. In a large study of colorectal surgery, laparoscopic colectomy was preferentially utilized despite the potential for a prolonged operative time, which was independently associated with an increased risk of postoperative pulmonary complications [37].

The clinical significance of composite meta-analyses of CCSs remains unclear, although various meta-analyses of CCSs in patients undergoing gastrectomy have been published [1–6, 38–40]. The value of meta-analyses of CCSs remains controversial, because CCSs often include groups of patients mismatched for background characteristics. To minimize bias in the present study, statistically mismatched CCSs were excluded. However, many of the included CCSs lacked detailed information on patients' characteristics, surgical procedures, and postoperative complications. A Korean RCT of distal gastrectomy in patients with early gastric cancer demonstrated that LDG was associated with lower incidences of several postoperative complications than was ODG [25], although a Chinese RCT of D2 lymphadenectomy in patients with advanced gastric cancer found no advantage of LDG with respect to postoperative complications [26]. Because LTG is a more difficult procedure than LDG, the results of RCT of distal gastrectomy should not be interpreted to be the same as those of total gastrectomy. A RCT comparing LTG with OTG is essential to clarify the advantages or disadvantages of LTG.

In conclusion, LTG was associated with significantly lower incidences of wound complications than was OTG in this meta-analysis of CCSs, although LTG had a slightly but not significantly higher incidence of anastomotic complications. The establishment of reliable anastomotic procedures is the most important concern in LTG.

## Supplementary Material

Figures of funnel plots of comparison; (A) anastomotic complications, (B) other intra-abdominal complications, (C) wound complications, (D) pulmonary complications, and (E) mortality.

## Figures and Tables

**Table 1:** Quality assessment of CCSs based on the Newcastle-Ottawa scoring system.

		Selection				Comparability^*∗*^ (maximum of 2 stars)	Exposure		
Author	Year	Is the case definition adequate?	Representativeness of the cases	Selection of controls	Definition of controls	Comparability on the basis of the design or analysis	Ascertainment of exposure	Same method of ascertainment	Nonresponse rate
Kim et al. [9]	2008	*∗*	*∗*			*∗∗*	*∗*	*∗*	*∗*
Topal et al. [10]	2008	*∗*	*∗*			*∗∗*	*∗*	*∗*	*∗*
Kawamura et al. [11]	2009	*∗*	*∗*			*∗∗*	*∗*	*∗*	*∗*
Kim et al. [12]	2011	*∗*	*∗*			*∗∗*	*∗*	*∗*	*∗*
Siani et al. [13]	2012	*∗*	*∗*			*∗∗*	*∗*	*∗*	*∗*
Bo et al. [14]	2013	*∗*	*∗*			*∗∗*	*∗*	*∗*	*∗*
Guan et al. [15]	2013	*∗*	*∗*			*∗*	*∗*	*∗*	*∗*
Hong et al. [16]	2013	*∗*	*∗*			*∗∗*	*∗*	*∗*	*∗*
Kim et al. [17]	2013	*∗*	*∗*			*∗∗*	*∗*	*∗*	*∗*
Kim et al. [18]	2013	*∗*	*∗*			*∗∗*	*∗*	*∗*	*∗*
Lee et al. [19]	2013	*∗*	*∗*			*∗∗*	*∗*	*∗*	*∗*
Shim et al. [20]	2013	*∗*	*∗*			*∗∗*	*∗*	*∗*	*∗*
Lee et al. [21]	2014	*∗*	*∗*			*∗∗*	*∗*	*∗*	*∗*
Lee et al. [7]	2015	*∗*	*∗*			*∗∗*	*∗*	*∗*	*∗*
Ramagem et al. [22]	2015	*∗*	*∗*			*∗∗*	*∗*	*∗*	*∗*

CCSs, case-controlled studies. ^*∗*^Select controls for patients' characteristics (age and gender) and clinical or pathological TNM classification.

**Table 2:** Summary of the studies in this meta-analysis.

Author	Published year	Duration of study	*N*	Tumor status	Extent of LND	Including splenectomy	Type of reconstruction	Reported matching factor^†^
Kim et al. [9]	2008	2004–06	60	pT1–3^*∗*^	D1+/D2	Yes	R-Y	1,2, 3,5, 7
Topal et al. [10]	2008	2003–06	60	pStage I–IV^*∗*^	D2	Yes	R-Y	1,2, 3,4, 5,6, 7
Kawamura et al. [11]	2009	2003–08	81	cT1	D2	No	R-Y	1,2, 3,4, 5,6, 7
Kim et al. [12]	2011	2009–10	190	cT1	D2-No.10	No	R-Y	1,2, 3,4, 5,6, 7
Siani et al. [13]	2012	2003–09	50	pStage I–III^*∗∗*^	D1+/D2-No.10,11d	No	R-Y	1,2, 5,6, 7
Bo et al. [14]	2013	2004–10	234	pT2-3^*∗*^	D1+/D2-No.10,11d	No	R-Y	1,2, 3,5, 6,7
Guan et al. [15]	2013	2007–10	97	cT1-2^*∗∗*^	D2	No	R-Y	2,5, 6,7
Hong et al. [16]	2013	2008–12	204	pStage I–III	D2	NR	NR	1,2, 3,4, 5,6
Kim et al. [17]	2013	2011	346	cT1–3 N0-2	D2-No.10	No	R-Y	1,2, 3,4, 5,6, 7
Kim et al. [18]	2013	2002–10	120	pSatge I–III^*∗∗*^	D1+/D2	No	R-Y	1,2, 3,4, 5,7
Lee et al. [19]	2013	2003–10	100	pStage I–IV^*∗*^	D1+	No	R-Y	1,2, 3,4, 5,6, 7
Shim et al. [20]	2013	2009–11	70	NR	D1+/D2	NR	R-Y	1,2, 7
Lee et al. [21]	2014	2006–09	84	cT1–3^*∗∗*^	D2-No.10,11d	No	R-Y	1,2, 5,6, 7
Lee et al. [7]	2015	2003–10	753	pT1^*∗*^	D1+	No	R-Y	1,2, 3,4, 5,6, 7
Ramagem et al. [22]	2015	2009–13	111	pStage I–III	D2	Yes	NR	1,2, 3,4, 5,6

LND, lymph node dissection; PSM, propensity score matching; R-Y; Roux-en-Y; NR, not reported.

^†^Factors showing no significant statistical difference between LTG and OTG: 1, age; 2, gender; 3, body mass index; 4, comorbidity or American Society of Anesthesiologists physical status; 5, clinical or pathological TNM classification; 6, extent of lymphadenectomy; 7, type of reconstruction. Some studies did not show statistical differences in several factors.

^*∗*^Based on the 6th version of TNM classification. ^*∗∗*^Based on the 7th version of TNM classification.
