# Supplementary material for: Postoperative Complications of Laparoscopic Total Gastrectomy versus Open Total Gastrectomy for Gastric Cancer in a Meta-Analysis of High-Quality Case-Controlled Studies
Source: Gastroenterol Res Pract. 2016 Nov 30;2016:2617903. doi: 10.1155/2016/2617903 (PMC5155090; doi:10.1155/2016/2617903)
Supplement: Supplementary file 1 — Figures of funnel plots of comparison; (A) anastomotic complications, (B) other intra-abdominal complications, (C) wound complications, (D) pulmonary complications, and (E) mortality. [file 2617903.f1.pdf]

Supplementary 1A

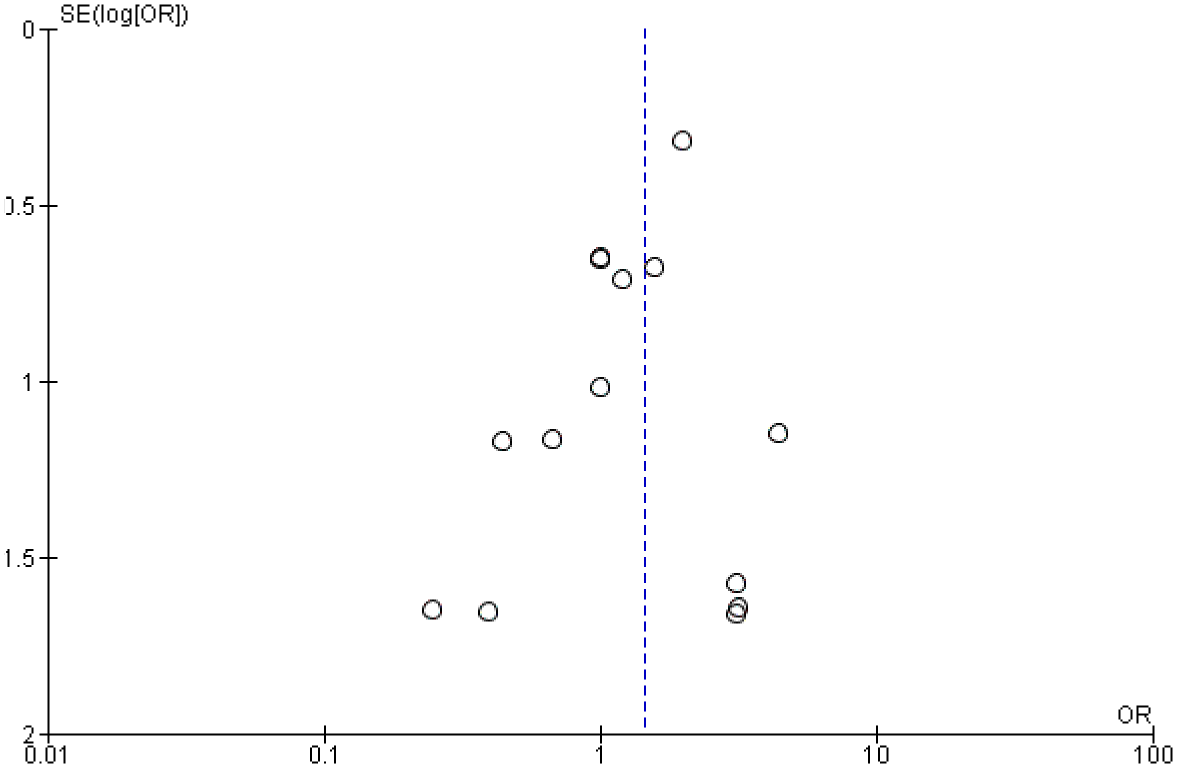


Supplementary 1B

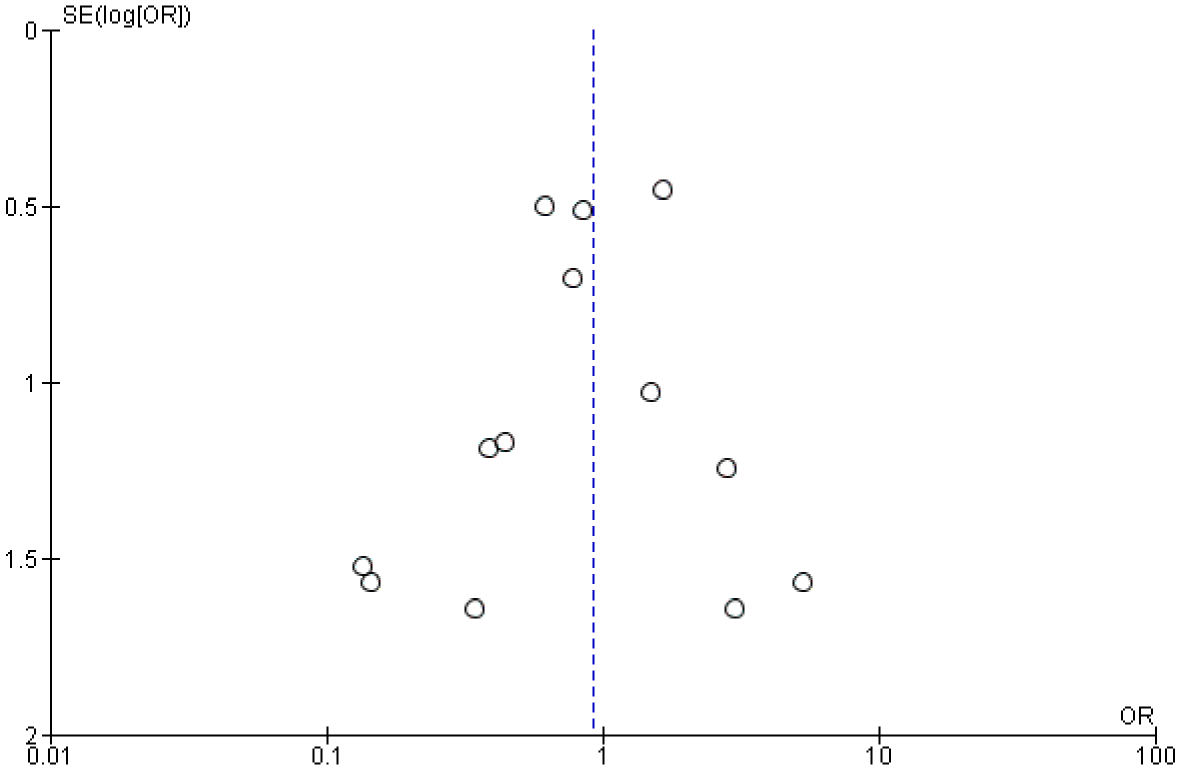

## Supplementary 1C

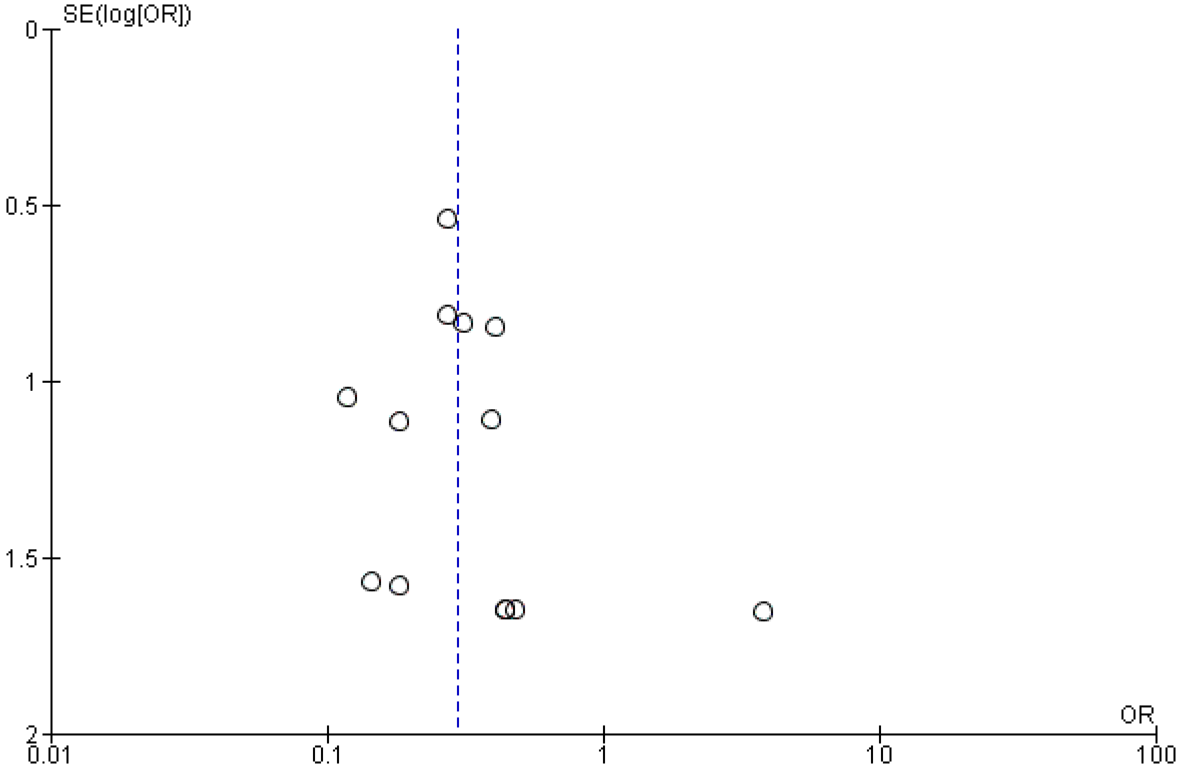


## Supplementary 1D

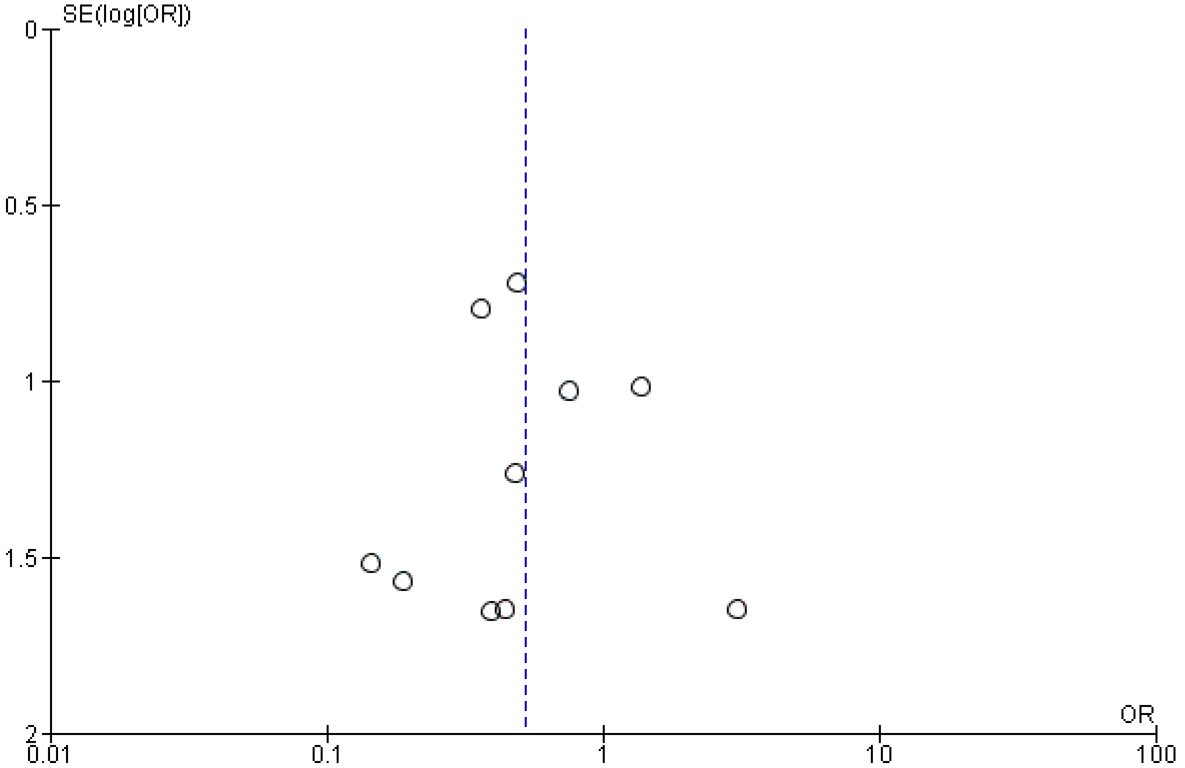

Supplementary 1E

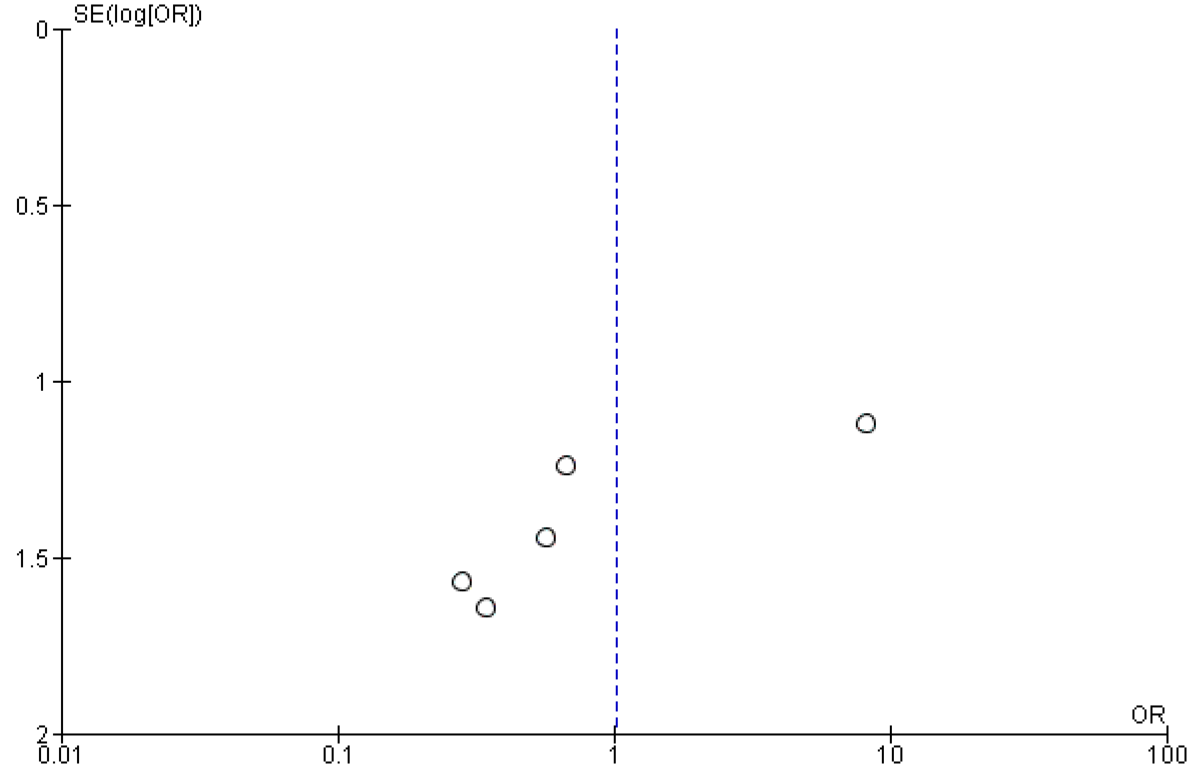


Supplementary 1. Figures of funnel plots of comparison; (A) anastomotic complications, (B) other intra-abdominal complications, (C) wound complications, (D) pulmonary complications, and (E) mortality
